# A Disinterested Appeal

**Published:** 1861-08

**Authors:** 


### A DISINTERESTED APPEAL.

WITH all the earnestness of our nature we urge on every subscriber and every casual reader of this JOURNAL to notice the article headed "Pay your Debts" in the last number; and when you have read it, have the manliness, the moral courage, the humanity, the Christian consistency, to make out a list of every indebtedness, and let not one single dollar remain in your purse overnight, unless it be that you are saving it to make up an amount to meet an imperative engagement. It is an absolute cruelty for any man who owes another, in these times, to permit money to lie idle in his hands. There are times when a single dollar may lift a mountain weight from the heart of a man who is worth thousands. A publisher narrated in our office, not long ago, an incident in reference to himself. "For a long time I had been preparing to meet a bank debt; it was a large sum to me—the only note in the world I had against me. Pay-day came, and I was not prepared. My wife and I had talked it over many times. She bravely denied herself even seasonable clothing, and arranged things in the kitchen so as to diminish the indispensable outlays to the smallest possible amount. Various persons who were owing had promised to do all in their power to help me in the emergency, for I went so far as to plainly state my case, and almost plead with them to do their best, their very best. But when the morning of the dreaded day came, the sun shone bright and beautiful, but there was no brightness nor beauty in it to me and mine. When the little children came to the breakfast-table there was such an ominous silence, that without any reference being made to the all-absorbing subject, even they seemed to feel the presence of an incubus. As is too often the case in such emergencies, one reliance fails, then another, and finally what a man can't do himself must remain undone. However, there at length remained only one dollar, a single dollar literally was wanting, after gathering up every penny in the house, even entrenching on the little savings of the children. Three o'clock was rapidly approaching, and the dreaded protest. What imaginings of ruin crowded my brain, coming and going with each successive turn of events! I could have borrowed the miserable dollar from any one of a multitude of friends; but I didn't borrow money, that involves reciprocities, magnified with Lord Ross's telescope; besides, to ask a friend to lend me a dollar, to have to confess I needed a single dollar so much, I could not stand it! A neighbor had owed me a dollar for a small book; it had been due a year; he had often told me to send for it, but I did not employ collectors, and to go myself to collect a dollar was "*infra dig.*" I could not stay in the house any longer. The mind wanted relief. I went out into the street as aimlessly as any loafing saunterer that ever disgraced manhood. Would you believe it, I met the very man who owed me the immensely-desired dollar, and before he came in hailing distance, he began to feel for his pocket-book, and with apologies for his remissness, he handed the amount! And what do you think I did? Why, like many a—I don't know what—before, I made out as if it was of no sort of consequence ; that any other time would have done as well—in fact, if it had never been paid, it was of no moment whatever ; and no actor on the stage could have exceeded the inimitable indifference with which I put out my hand to receive the rag. But as soon as he turned the corner, didn't I clutch that paper-dollar ! didn't I heel it down to Wall-street at 2 : 40—and "better," by a baker's dozen ? Didn't I take up that note, and vow most religiously that I never would give a note in hand again the longest day I lived ? Nor have I yet. But every time I think of it there is a sinking within my bosom, and an abasement at the remembrance that I was still full of poor weak human nature in that "I made believe" I didn't care about that contemptible dollar. Reader, there are multitudes of similar cases taking place in New-York and other large cities and towns every day. If every subscriber to a newspaper or magazine would but have the honesty to remit a due subscription the instant he lays down this paper, nay, more, if every such person who has the amount in his pocket, or at home, would do this at this juncture, an amount of depression, if not of agonizing anxiety, would be removed from a large class of industrious, hard-working, indulgent, and honorable publishers of newspapers, of books, and of magazines, that is utterly incalculable. Not a solitary subscriber owes us a dollar ; on the contrary, we owe them four more Journals ; but we are urging a plea for our exchanges, some of whom have stopped, others are in a deadly drag, and many more must fall into the same condemnation, some of them losing the products of the labor of a lifetime ; and all this because the men whom they have done so much to amuse and instruct and gratify, withhold the pittance of a dollar or two or three, which they could certainly pay, if they had but the will. Shame, a burning shame, to all such !

The order of payment is of great practical importance. It is a ten-fold economy of happiness and health to pay ten debts averaging a dollar each, than to pay one of ten dollars ; for ten persons are gratified, ten holes are stopped, ten chances of being dunned are removed instead of one, ten annoyances are got rid of instead of one ; for what is a greater annoyance, a greater jar on a sensitive mind than to be dunned for a dollar when there is not a penny in the pocket ? You feel mean because you are so poor, and meaner still from the consciousness that your neighbor has found out that you can not pay a contemptible dollar, while if you know that he really needs it, mortification and regret are added to the catalogue. The smallest debts should be paid first, on the presumption that the smaller the debt, the poorer is your creditor, the less his ability to borrow, in case he is disappointed in getting what you owe him, and the less can he afford the time required in calling on you.

